# High-risk clonal groups of avian pathogenic *Escherichia coli* (APEC) demonstrate heterogeneous phenotypic characteristics *in vitro* and *in vivo*

**DOI:** 10.1080/21505594.2025.2546682

**Published:** 2025-08-13

**Authors:** James R. G. Adams, Huijun Long, Charlotte A. Birdsall, Kamran Qureshi, Emma King, Sara Perez, Keith Warner, Shahriar Behboudi, Roberto M. La Ragione, Jai W. Mehat

**Affiliations:** a Department of Comparative Biomedical Sciences, School of Veterinary Medicine, Faculty of Health and Medical Sciences, University of Surrey, Guildford, UK; b Avian Immunology Group, The Pirbright Institute, Woking, UK; c Discipline of Microbes, Infection and Immunity, School of Biosciences, Faculty of Health and Medical Sciences, University of Surrey, Guildford, UK; d Poultry Health Services, York, UK; e Avara Foods Ltd, Brackley, UK; f Bristol Veterinary School, University of Bristol, Langford, UK

**Keywords:** APEC, avian pathogenic, *Escherichia coli*, sequence types, cell culture, *Galleria mellonella*

## Abstract

Avian Pathogenic *Escherichia coli* (APEC), a major bacterial pathogen of poultry, comprises a diverse range of high-risk clonal groups. However how these lineages interact with avian host cells remains poorly characterized. This study examined the ability of key APEC clonal groups to adhere to, invade, and survive within avian host cells, alongside assessing their virulence in the *Galleria mellonella* infection model. Genomic analysis of APEC from a UK turkey colibacillosis outbreak identified ST-101 as the dominant clonal group, carrying numerous virulence factors. ST-101 was compared to other high-risk APEC clonal groups (ST-23, ST-140, ST-95, ST-117). Utilizing *in vitro* cell culture models, APEC isolates displayed comparable adhesion to 8E11 chicken epithelial gut and HD11 chicken macrophage cell lines. APEC ST-95, ST-101, and ST-140 demonstrated increased invasion of 8E11 cells, and intracellular survival within HD11 macrophages, relative to ST-23 and ST-117, suggesting pronounced phenotypic differences between clonal groups. However, in HD11 cell assays, no difference in magnitude of elicited immune response was observed between lineages, indicating differing intracellular survival was not a result of immune response modulation. *In vivo* virulence in the *Galleria mellonella* infection model was also observed to differ between APEC genotypes, with ST-117 inducing the highest mortality, despite the comparatively lower epithelial invasion and intramacrophage survival for other lineages. Collectively, this suggests that diverse APEC genotypes have distinct phenotypic profiles *in vitro* and *in vivo*. These results highlight the need for intervention strategies that can simultaneously target a broad range of pathogenic lineages.

## Introduction

*E coli* are typically a commensal organism of the vertebrate gut, exhibiting a mutualistic relationship with its host. This role is fulfilled by preventing pathogen colonization by competitive exclusion [1], priming the immune system against infection [2,3], and producing vitamins for the host [4]. However, a minority of genetic lineages are responsible for a variety of extra-intestinal pathologies, most commonly manifesting as cellulitis, or respiratory infections that progress to systemic colisepticaemia. Pathogenic, or pathobiont lineages of *E. coli*, may arise through the horizontal acquisition of virulence determinants, which are often independent events in disparate phylogenetic backgrounds. This has resulted in the repeated emergence of distinct pathogenic lineages capable of eliciting disease in healthy hosts [5,6]. *E*. *coli* lineages are frequently categorized by multi-locus sequence typing (MLST) and as distinct sequence types (STs). Primary pathogenic lineages are broadly divided into intestinal pathogenic *E. coli* (InPEC) (comprising STc11, STc29, ST16, and ST17) and extra-intestinal pathogenic *E. coli* (ExPEC) (STc131, STc95, STc73, and STc69) pathotypes [6]. Avian pathogenic *E. coli* (APEC) is the causative agent of avian colibacillosis [7], a range of localized and systemic infections that collectively cause significant financial, and animal health and welfare issues stemming from increased bird mortality, diminished egg production, and reduced hatchability, affecting every production system in all farmed species of poultry. APEC may be transmitted vertically through the transovarian route, resulting in contamination of the eggshell surface, or acquired post-hatch from environmental exposure within hatcheries; with both routes posing a risk of early chick mortality. Within poultry flocks, horizontal transmission of APEC can occur via inhalation of aerosolized particles, contact with contaminated feces, or through ingestion of polluted water sources, facilitating rapid dissemination of pathogenic lineages.

Several approaches for typing APEC have been used including serotyping, and the presence of particular virulence genes [8]. However high genome diversity and plasticity of implicated isolates renders approaches based on the number and constellation of virulence genes insufficient for differentiating high-risk genotypes from commensal *E. coli* [8–12]. Multiple sequence types are associated with APEC globally, however STc-23, ST-95, ST-117, ST-140, and ST-428/429 have been identified as high-risk clonal groups [9,13–15]. Multiple strategies for the control of APEC have been developed, including vaccination aimed at targeting the O78 serotype that belongs to the STc-23 and ST-117 lineages [9]. However, heterologous protection from vaccination can be inconsistent [16–20]. Moreover, the impact of vaccination on horizontal transmission rates of virulence-associated plasmids among avian-associated *E. coli* is unknown [21–23].

Plasmids can facilitate the rapid dissemination of genes within microbial environments, driving genetic divergence and impacting bacterial fitness [24,25]. The Colicin V (ColV) plasmid is over-represented in APEC [23,26], and encodes a number of virulence-related genes such as adhesins, toxins, or iron acquisition systems [27,28]. ColV/IncF plasmids are widespread in *E. coli* and APEC, with one study identifying the ColV genes *cvaA* and *cvaB* present in the majority of turkey clinical isolates [29]. Considering epidemiological studies implicate specific STs in the majority of outbreaks, it is clear that these virulence plasmids work in concert with defined phylogenetic backgrounds to mediate disease occurrence. However, the exact contribution of ColV to pathogenicity and the relative importance of these plasmids in the context of differing *E. coli* genetic backgrounds is not fully understood [8,30]. This highlights the need to unravel the precise genetic determinants which contribute to APEC virulence and elucidate how these may differ between high-risk APEC clonal groups.

This study aimed to characterize the interaction of APEC with avian epithelial cells and macrophages. In this study, we identify the *E. coli* ST-101 sequence type as a highly prevalent lineage implicated in a recent colibacillosis outbreak in turkey poults. Comparison of the pathogenic potential of ST-101 relative to APEC isolates belonging to the predominant, high-risk clonal groups demonstrated that APEC clonal types differ in their capacity to survive intracellularly in avian macrophages and epithelial cells. Finally, using the *in vivo Galleria mellonella* infection model, we demonstrate that APEC isolated from different sequence types vary in their pathogenic potential.

## Methods

### Isolation and identification of *E. coli* implicated in a colibacillosis outbreak in Turkey poults

A total of 91 *E. coli* isolates were collected from turkeys housed on a single commercial farm in the UK experiencing an increase in 7-day bird mortality between May and September 2019. All birds from which *E. coli* was collected either died naturally or were humanely euthanized due to clinical signs and symptoms of colibacillosis, or for population management. At routine *post-mortem* examination of 113 sick and healthy birds, swabs of lesions on the heart and liver, and swabs of cecal contents were inoculated onto MacConkey Agar No. 3 (Oxoid, Basingstoke, UK), and the bacterial species identified by replica plating onto CHROMagar Orientation (Trafalgar Scientific, UK). 91 pink-red colonies indicating pure cultures of *E. coli* were ultimately obtained and these were selected for downstream analyses (Table S1).

### DNA extraction and sequencing of outbreak APEC isolates

For DNA extraction of *E. coli* isolates, a single representative colony was used to inoculate 10 mL of fresh LB broth in a sterile 50 mL Falcon tube before aerobic incubation at 37°C with shaking at 225 RPM, for 18 hours. DNA was extracted from a broth culture of each isolate, using a Wizard Genomic DNA purification kit (Promega, Wisconsin, United States) following the manufacturer’s instructions. DNA quantity, integrity (1.8–2 260/280 nm absorbance ratio), and purity (2–2.2 260/230 nm absorbance ratio) were assessed using a Biodrop (DKSH, Zurich, Switzerland), and stored at −20°C. Extracted DNA was sequenced using a commercial service (MicrobesNG, Birmingham, UK) short read 250 base pair (bp) paired end reads were generated on an Illumina MiSeq with a minimum of 30x coverage. Representative isolates SAP4026 and SAP4027 were selected for hybrid sequencing to generate complete ColV plasmid sequence. This combined 250 bp paired end Illumina MiSeq reads with MinION (Oxford Nanopore) long reads to generate complete assemblies.

### Genomic assembly, annotation, phylogenetic reconstruction, and multi-locus sequence typing

Raw sequence reads were assembled into contigs using Shovill (https://github.com/tseemann/shovill), followed by annotation using Prokka (https://github.com/tseemann/prokka), with a minimum contig length of 200. The 91 genome assemblies were used to construct a phylogenetic tree using Parsnp v1.2 with a k-mer length of 15 and set to autopick the reference genome (https://github.com/marbl/parsnp) and visualized using iTOL (https://itol.embl.de/). Phylogroups and serotypes were determined using the EcOH database and Enterobase [31]. Abricate (https://github.com/tseemann/abricate) was used to screen genome assemblies for virulence factors using a custom abricate database comprising common *E. coli* and APEC virulence genes [32]. The multi-locus sequence types (MLST) of APEC genomes were determined using the *mlst* software (Seemann T, mlst, Github https://github.com/tseemann/mlst), using the Warwick scheme [33]. RFplasmid v0.0.18 was used to classify each contig as plasmid- or chromosome-like based on the basis of characteristic genes and pentamer frequencies. This software employs a random forest classifier to attribute votes ranging from 0–1 to each contig, allowing discrimination between plasmid and chromosome contigs. This allowed identification of putative ColV plasmid genes [34]. To examine the extent of genetic similarity between ColV plasmids identified within this study and high-virulence gene density (pAPEC-O2-211A-ColV assession number: CP030791.1 and pAPEC-p10_578_1 assession number: CP087565.1), and low-virulence gene density (pCh101 assession number: CP127318.1) ColV plasmids were selected from the NCBI database using Clinker v0.0.31 [35].

### Preparation of bacteria for use in cell culture infection assays

APEC isolates (Table 1) were streaked onto MacConkey Agar No. 3 (Oxoid, Basingstoke, UK) while *S*. Typhimurium SL1344 was streaked onto nutrient agar (Oxoid, Basingstoke, UK), before incubation at 37°C, aerobically for 18 hours. For broth cultures, a single representative colony of each isolate was used to inoculate 10 mL of fresh LB broth in a sterile 50 mL falcon tube before aerobic incubation at 37°C with shaking at 225 RPM, for 18 hours.

**Table 1.** List of bacterial isolates used in this study for cell culture assays. This panel is comprised of representative APEC isolates from the major sequence types implicated in colibacillosis outbreaks in Europe (ST-23, ST-117, and ST-95), and the predominant serotypes are represented. Two ST-101 isolates from the colibacillosis outbreak in turkey poults are also included in this panel.

Isolate Code	Phylogroup	Serotype	Multi-locus Sequence Type	Source of isolation	Country of origin
SAP 503	C	O78:H4	ST23	Layer	UK
SAP 482	C	O78:H4	ST23	Layer	UK
SAP 557	B2	O2:H5	ST140	Layer	UK
SAP 551	B2	O2:H5	ST140	Layer	UK
SAP 487	B2	O45:H7	ST95	Layer	UK
SAP 537	B2	O18:H7	ST95	Layer	UK
SAP 631	G	O78:H4	ST117	Broiler	UK
SAP 641	G	O78:H4	ST117	Broiler	UK
SAP 494	G	O24:H4	ST117	Layer	UK
SAP4026	B1	088:H8	ST101	Turkey	UK
SAP4027	B1	O88:H8	ST101	Turkey	UK
SAP16			*Salmonella* Typhimurium (SL1344)		

Previously characterized APEC isolates and *Salmonella* Typhimurium SL1344, an established positive control for cellular invasion and intracellular survival, that consistently and reproducibly invades cells and yields intracellular bacterial counts [36], were also used as comparator isolates within this study (Table 1). Selection of APEC lineages for inclusion within the panel was based on published literature on APEC prevalence [9,13,37–41].

### Growth and maintenance of cell lines

The chicken gut epithelial cell line 8E11 (Micromol, Germany) [42] and chicken macrophage-like cell line HD11 [43] were used within this study. 8E11 cells were maintained in Dulbecco’s Modified Eagle Medium/Nutrient Mixture (DMEM) F-12 supplemented with glutamine (ThermoFisher, Paisley, UK), 10% fetal bovine serum (FBS) (ThermoFisher, Paisley, UK), and 1% Penicillin/Streptomycin (ThermoFisher, Paisley, UK) at 37°C with 5% CO_2_. The HD11 cell line was maintained in Roswell Park Memorial Institute (RPMI) 1640 Medium (ThermoFisher, Paisley, UK) supplemented with 5% FBS, 5% chicken serum (CS) (ThermoFisher, Paisley, UK) and 1% Penicillin/Streptomycin at 37°C with 5% CO_2_. When 80% confluency was reached, cells were detached using 0.25% trypsin (ThermoFisher, Basingstoke, UK) and used to seed 24 well plates (Greiner, Stonehouse, UK) at a concentration of 2 × 10^5^ cells/ml.

### Bacterial intracellular survival assay

8E11 or HD11 cells were seeded at 2 × 10^5^ cell/mL in a total of 1 mL media per well in 24 well tissue culture treated plates. Plates were incubated for 24 hours at 37°C with 5% CO_2_ prior to assay to ensure adherence.

8E11 cells were challenged with bacteria at a multiplicity of infection (MOI) of 100 (2 ×10^7^ CFU/mL) and HD11 cells were challenged with an MOI of 10 (2 ×10^6^ CFU/mL) in triplicate before centrifugation at 4°C for 3 minutes at 300 ×g. Plates were incubated for two hours before aspiration of the media and washing of the plates twice with PBS. To determine bacterial association to eukaryotic cells, media was removed, and cells were washed twice with PBS before 1 ml of 1% Triton 100X (Merck, Kenilworth, UK) in PBS was added to the wells. The solution was gently pipetted to disrupt the eukaryotic cell membrane and resuspend the associated bacteria. The CFU/ml of the inoculant was calculated using the Miles and Misra technique [44]. Briefly, cell lysate was serial diluted 10-fold in sterile PBS and 20 µL of each dilution was dropped in triplicate onto nutrient agar before incubation for 16 hours at 37°C aerobically. Colonies were then counted and colony forming units (CFU) per mL calculated. To determine intracellular survival, a gentamicin protection assay was performed by the addition of 1 mL cell culture media supplemented with 100 µg/mL gentamicin and incubation for an additional 2, 4, or 16 hours. Cell supernatant was then aspirated and HD11 were washed twice with warm PBS and 1 mL 1% triton X in PBS was added to lyse cells, before viable counts were determined [44].

### Determination of nitric oxide and reactive oxygen species production following bacterial challenge

HD11 cells were seeded in 24 well tissue culture treated plates in at 2 × 10^5^ cell/mL in a total of 1 mL media per well at least 24 hours prior to assay to ensure adherence. HD11 cells were challenged in triplicate with bacterial isolates prepared as previously described at a MOI of 10. Cells were then centrifuged at 4°C for 3 minutes at 300 ×g before incubation at 41°C with 5% CO_2_ for 1 hour.

To determine nitric oxide (NO) production, cell supernatant was removed and replaced with 1 mL of RPMI supplemented with 5% FBS 5% CS, and 100 µg/mL gentamicin and incubated for a further four hours. The cell supernatant was transferred to a 1.5 mL microcentrifuge tubes and centrifuged at 2500 ×g for 10 minutes at 4°C to pellet cell debris. Following this, 150 µL of the supernatant was mixed with 130 µL ddH_2_O and 20 µL Griess reagent (ThermoFisher, Paisley, UK) in triplicate in a 96 well plate (Greiner, Stonehouse, UK). The plate was incubated at room temperature, protected from light for 30 minutes and absorbance was read at 548 nm on a TECAN Spark plate reader and nitrate concentration (the auto-oxidation product of NO) was determined using a calibration curve.

To determine ROS production, supernatant was removed and replaced with 1 mL of RPMI supplemented with 5% FBS 5% CS, 100 µg/mL, and 20 µM DCFA (D399, ThermoFisher, Paisley, UK). The plate was incubated in the Clariostar plate reader (BMG Labtech, Ortenberg, Germany) at 41°C and 5% CO_2_ for a further ten hours with fluorescence at 495 nm excitation and 529 nm emission measured every 15 minutes.

### Galleria *mellonella* infection model

*Galleria mellonella* larvae in the fifth or sixth instar stage were purchased from Livefoods UK (Sheffield, UK). Larvae were maintained at 17°C on woodchips and used in assays within two weeks of delivery and handled using blunt-nosed forceps. Healthy *Galleria* larvae, weighing between 180 and 260 mg [45], were examined to ensure uniform coloring (white/cream) and which had the ability to right themselves after being inverted were separated into groups of 10 in duplicate. APEC isolates SAP482, SAP487, SAP631, or SAP4026 (Table 1) were streaked onto MacConkey agar plates and incubated aerobically at 37°C for 18 hours before colonies were suspended in PBS at a OD_600_ of 0.125 before diluting to reach a final concentration of 1x10^3^ CFU per 10 µL. Larvae were then infected as described previously [46,47], in the top right proleg with either 10 µL of bacterial suspension containing of 1x10^3^ CFU or 10 µL sterile PBS, using a Hamilton 26S Microliter syringe (Merck, Kenilworth, UK). Following injection, larvae were placed on 90-mm filter paper in an inverted Petri-dish and incubated for 72 hours at 37°C. Mortality was determined every 24 hours by observing melanization and ability to respond to physical stimulation [46].

## Results

### APEC ST-101 are the most prevalent sequence-type implicated in a colibacillosis outbreak and encode a high density of APEC-associated virulence determinants

Between May and September 2019, a notable increase in 7-day mortality was observed among turkey poults housed on a single commercial turkey farm in the UK. While baseline mortality during the first week post hatch typically was 1%, this figure rose significantly to 4% during the affected period. *Postmortem* examinations conducted as part of routine veterinary practice and surveillance resulted in the recovery of 91 *E. coli* isolates from poults exhibiting clinical signs consistent with colibacillosis. This elevated mortality was attributed to an outbreak of APEC.

**Figure 1.**
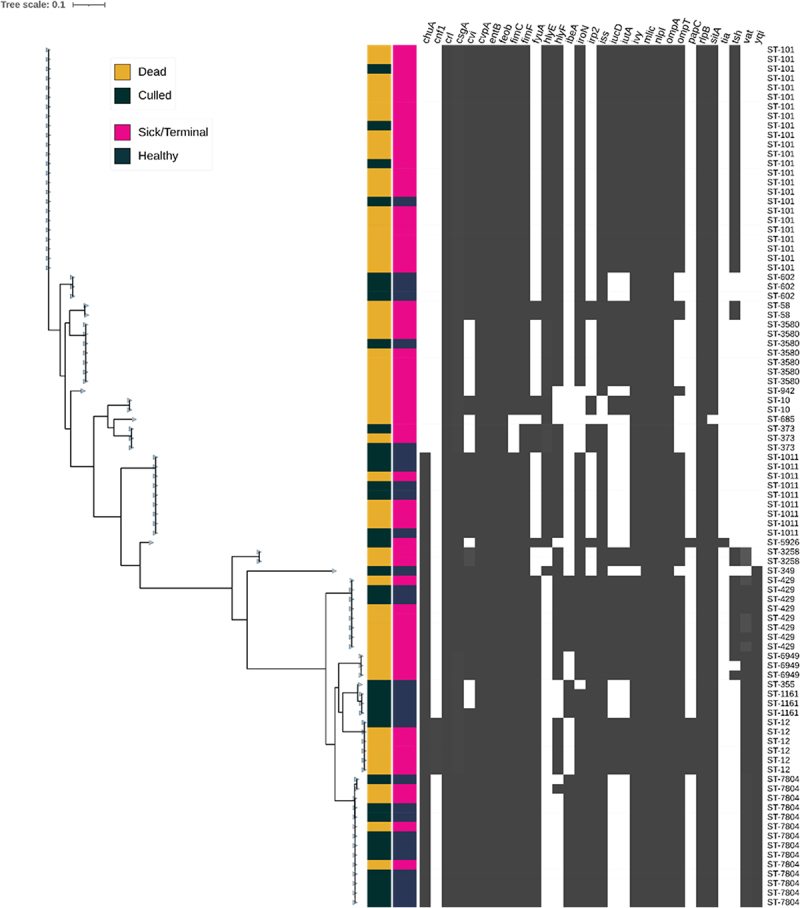
Phylogenetic reconstruction of 91 *E. coli* genomes (Table S1) recovered from a colibacillosis outbreak in Turkey poults. Whole genome sequences of *E. coli* isolated from the heart, liver, and caeca of turkeys suspected of colibacillosis were used to reconstruct core-genome phylogeny using ParSNP [48]. Health-associated metadata of bird fate (dead or culled) and health status (sick/terminal or healthy) denoted by the colour strip to the right of the cladogram. Presence of encoded APEC-associated virulence genes determined using a custom abricate (https://github.com/tseemann/abricate) database using an 80% minimum sequence identity threshold. The presence or absence of a virulence gene within the bacterial genome is denoted by a grey box in the heatmap (grey=present, white=absent). Sequence type (ST) was established using Warwick classification scheme (Seemann T, *mlst*, Github https://github.com/tseemann/mlst) and denoted to the right of the heatmap.

Outbreak isolates were sequenced and determined to belong to 18 distinct sequence types (STs), with ST-101 observed to be dominant within the outbreak (26.3% of all isolates) (Figure 1). Isolates belonging to ST-7804 were second most prevalent, accounting for 15.3% of all APEC recovered. Comparison of metadata also revealed an association between disease state and sequence type. Of the 25 isolates belonging to ST-101, 23/25 (92%) were recovered from sick birds. Meanwhile, only 4/12 ST-7804 isolates (33.3%) were isolated from diseased or dead birds. Isolates identified as ST-3508 were isolated from “sick/terminal” birds in 85% (6/7) of cases observed. Together, this highlights ST-101 as the dominant clone within the outbreak and a potentially increased capacity to cause disease within birds.

**Figure 2.**
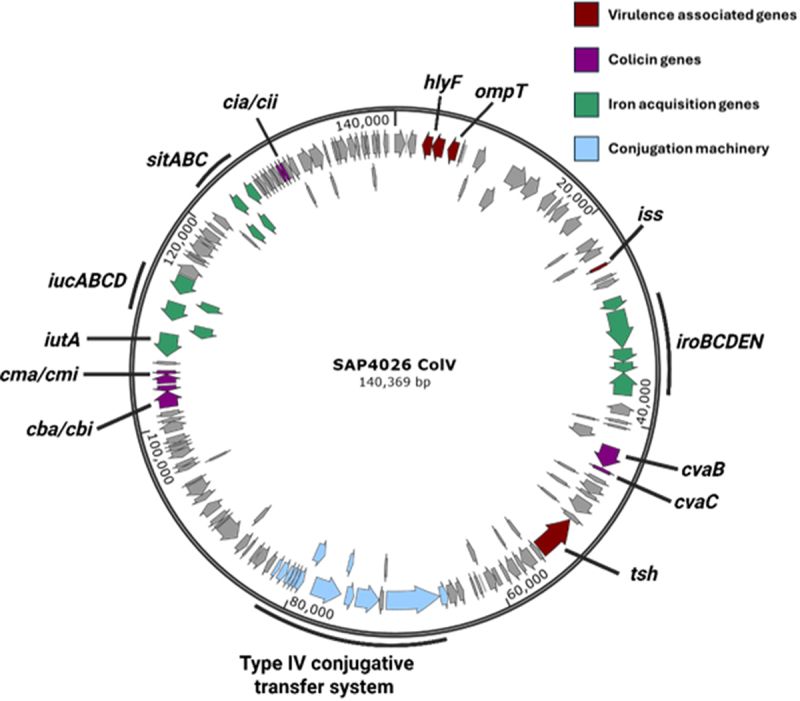
Plasmid map of ColV plasmid harboured by ST-101 SAP 4026. Genes identified by Bakta (https://github.com/oschwengers/bakta) annotation of circular plasmid contig determined through hybrid sequencing. Dark red = virulence associated genes. Purple = colicin genes. Green = iron acquisition genes. Blue = conjugation machinery. Created with SnapGene software (www.snapgene.com).

Due to its dominance within the outbreak and association with bird mortality and morbidity, ST-101 APEC, encoding the O88:H8 serotype antigens, were characterized further. *In silico* screening of virulence genes revealed a highly conserved virulence profile, encoding genes associated with colonization and adhesion (*crl, csgA*, *fimC*, and *fimF*), iron acquisition systems (*iutA, iucC, iucD*, and *sitA*), protectins (*iss, mliC, ompT*, and *OmpA*), and toxins (*hlyE, hlyF*, and *vat*) [49]. To investigate the distribution of these genes between the chromosome and plasmid of the ST-101 isolates hybrid short and long read sequencing of an isolate designated as SAP4026 was performed. Five closed contigs were identified, consisting of the bacterial chromosome and four plasmids. One plasmid identified as a Colicin V plasmid common to APEC encoded a large density of virulence associated genes (Figure 2). This included a range of iron acquisition genes including *iroBCDEN, sitABC, iucABCD*, and *iutA*. Colicin A, B, C, and M genes were also identified on the same plasmid, alongside temperature-sensitive hemagglutinin *tsh*, increased serum survival protein *iss*, outer membrane protease family protein *ompT*, and *hlyF*. Type IV conjugation machinery were also identified within the plasmid, suggesting a capacity for horizontal transfer.

**Figure 3.**
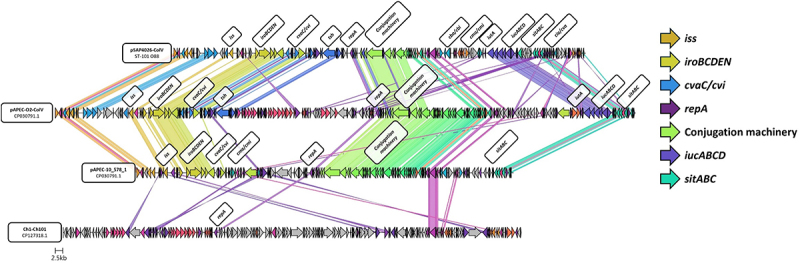
Gene cluster comparison of pSAP4026-ColV with previously reported plasmids from avian pathogenic and commensal *E. coli*. Figure generated using the clinker and cluster map software [35] with identity threshold set at 0.3. Gene clusters demonstrating similarity greater than the threshold share the same colour code. Additional labelling performed in biorender (www.biorender.com). Plasmid sequences were selected from comparison from the NCBI database; APEC-O2-211A-ColV (Assession number: CP030791.1), pAPEC-p10_578_1 (Assession number: CP087565.1), and pCh101 (Assession number: CP127318.1) accessed through GenBank.

The ColV plasmid identified within ST-101 was compared to ColV plasmids previously identified within bacteria from chickens, selected from the NCBI database to represented high- and low virulence gene density. This revealed shared identity with previously reported APEC-associated ColV plasmids, pAPEC-O2-211A-ColV (Assession number: CP030791.1) and pAPEC-p10_578_1 (Assession number: CP087565.1), with clusters identified as *iss, ironBCDEN, sitABC*, and type IV conjugation machinery observed to be shared. However, extensive variation was observed between the pSAP-4026 ColV plasmid and the similarly sized pCh101 (Assession number: CP127318.1) previously identified within commensal avian *E. coli* (Figure 3). This typifies the variability in ColV plasmids amongst APEC, in terms of gene content and synteny.

Collectively, characterization of the colibacillosis outbreak identified ST-101 O88:H8 as the dominant clonal lineage which was typically associated with the development of disease in birds and harbored multiple plasmid virulence factors.

### APEC ST-101, ST-95, and ST-140 exhibit higher levels of invasion of 8E11 chicken gut epithelial cells than ST-23 and ST-117

Within the colibacillosis outbreak, multiple APEC genotypes were isolated from lesions of diseased bird, however it was dominated by the ST-101 clonal group. ST-101 APEC have previously been reported within poultry in multiple countries [13,32,39,50,51], in addition to being associated with recent colibacillosis outbreaks in Denmark [52]. Yet, despite the growing prominence of this lineage, the phenotypic profile of ST-101 and how it may differ from high-risk clonal groups remains poorly understood. Therefore, ST-101 APEC isolated from this outbreak in turkeys was compared to APEC lineages frequently associated with avian colibacillosis in the UK and Europe [9], using isolates previously identified in colibacillosis outbreaks in chickens (Table 1) using the 8E11 chicken gut epithelium *in vitro* model. The 8E11 cell line was used as a model to quantify the ability of APEC to adhere to and invade avian cells. Isolates were compared to *S*. Typhimurium, an intracellular pathogen capable of high levels of adherence and invasion of eukaryotic cells, as a benchmark.

**Figure 4.**
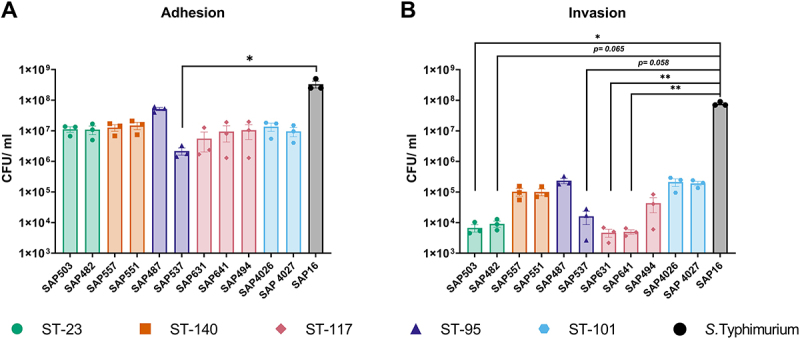
Comparison of ability of APEC isolates to (A) adhere to and (B) invade the 8E11 cell line. Quantification of adhesion of APEC isolates determined following challenge with MOI 100 bacterial inoculum followed by two hours incubation at 41°C prior to washing and lysing of eukaryotic cells. Invasion assayed by the inoculation of cells for two hours followed by the addition of cell culture media containing gentamicin for a further two hours, before washing and lysis of eukaryotic cells. Bacterial viability determined by the Miles and Misra method. Experiments performed independently three times, with triplicate wells used for each isolate. Data shows average CFU/ml ± SEM. Significance determined by Kruskal Wallis statistical test, followed by post-hoc Dunn’s test of multiple comparisons compared to SAP16 with significance denoted by *= *p* ≥ 0.05, ** = *p* ≤ 0.01. ST-101 APEC demonstrated increased intracellular survival within HD11 chicken macrophage cells compared to ST-117 APEC.

Adhesion to cellular surfaces prevents mechanical clearance and confers a selective advantage for pathogenic bacteria [53]. Here, all APEC isolates demonstrated the ability to adhere to the 8E11 cell line, with no significant difference (*p* ≤0.05) observed between APEC lineages (Figure 4A). Comparison to the invasive pathogen *S*. Typhimurium saw no significant difference in adherence capacity of APEC isolates, with the exception of APEC ST-95 O18 where adherence was significantly (*p* ≥0.05) reduced compared to *S*. Typhimurium. In addition to adhesion, *E. coli* and other Gram-negative pathogens have been observed to invade host cells, allowing intracellular replication and reduced competition for nutrients [53,54]. Ability of APEC isolates to invade 8E11 cells showed significant (*p* = 0.0013) variation between APEC lineages (Figure 4B). Invasive ability compared to *S*. Typhimurium was also observed to be significantly lower in the ST-23 SAP503 (*p* = 0.018) and SAP482 (*p* = 0.065), the ST-95 isolate SAP537 (*p* = 0.058), and ST-117 SAP631 and SAP 641 (*p* = 0.008). In contrast, isolates belonging to ST101, ST-140, and the ST-95 SAP487 isolate demonstrated a greater degree of invasion, though still lower than *S*. Typhimurium (*p* > 0.999). Intra-sequence type variations were also observed within isolates with different O-antigen serotypes, with SAP487 O45 and SAP537 O18 demonstrating both the highest and lowest invasive ability, respectively. All APEC lineages demonstrated similar (*p* ≤0.05) growth rate in 8E11 cell culture media (Figure S1), indicating that differences in bacterial load recovered was not a result of increased growth. Collectively, this suggests that genotype defines the capacity of APEC to invade gut epithelial cells.

**Figure 5.**
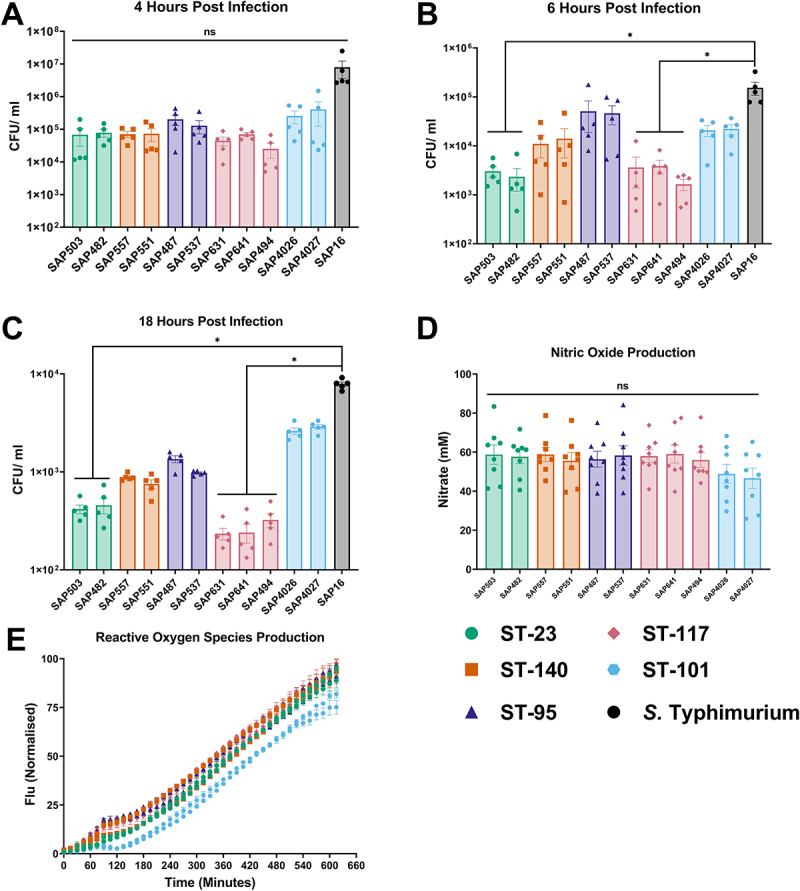
Comparison of intracellular survival of APEC isolates in HD11 chicken macrophage cells at (A) 4 hours post infection, (B) 6 hours post infection, and (C) 18 hours post infection. HD11 cells challenged with a MOI 10 bacterial inoculum and incubated for two hours at 41°C. Intracellular survival was determined by the addition of gentamicin containing media for the described incubation period and quantification of bacteria viability. Experiments performed independently five times, with triplicate wells used for each isolate. Data shows average CFU/ml ± SEM. Production of (D) NO determined by Griess assay six hours post-infection. Experiment performed independently eight times with triplicate wells used for each isolate. Data shows average nitrate concentration ± SEM. Production of (E) ROS production by addition of DCFA and measurement of fluorescence intensity for 10 hours post infection. Experiment performed independently three times with triplicate wells used for each isolate. Data shows average fluorescence intensity (flu) ± SEM. Significance determined by Kruskal Wallis statistical test *** = *p* ≤ 0.001, followed by post-hoc Dunn’s test of multiple comparisons, with differing letters denoting significance (*p ≥* 0.05).

Macrophages are the first line of defense following infection, phagocytose and killing invading pathogens. However, many pathogens, including some *E. coli* [55], have also evolved to resist phagocytosis and intracellular killing by macrophages, thereby aiding systemic infection. The *in vitro* HD11 avian macrophage cell line was used to compare the ability of APEC lineages to survive intracellularly. At four hours post-infection, all APEC sequence types exhibited comparable (*p* = 0.1308) intracellular survival within chicken macrophages (Figure 5A). However, significant (*p* < 0.001) differences between APEC sequence types were present at six hours (Figure 5B) and 18 hours post infection (Figure 5C). Comparison of intracellular survival to that of *S*. Typhimurium revealed trends between sequence types, alike those observed within 8E11 cell invasion (Figure 4). At both six hours and 18 hours post infection, bacterial survival was significantly reduced in ST-23 isolates SAP503 (*p* > 0.001) and SAP482 (*p* > 0.01), as well as ST117 isolates SAP631 (*p* > 0.01), SAP641 (*p* > 0.001), and SAP494 (*p* > 0.001) compared to *S*. Typhimurium. Meanwhile, isolates belonging to ST-140, ST-95, and ST-101 demonstrated high intracellular survival, comparable (*p* ≤0.05) to *S*. Typhimurium. Adhesion to HD11 chicken macrophage was confirmed to be equivalent (*p* ≤0.05) between APEC isolates tested, suggesting that changes in intracellular survival were not a result of reduced phagocytosis (Figure S2).

Nitric oxide (NO) and reactive oxygen species (ROS) contribute to antimicrobial activity and are frequently used as an indicator of the magnitude of inflammatory immune response [56]. Nitric oxide production was induced by all isolates following exposure and incubation for 18 hours, with no significant (*p* ≤0.05) differences observed (Figure 5D). Similarly, ROS production was induced following infection of HD11 cells with all APEC lineages (Figure 5E). Macrophages infected with ST-101 APEC exhibited delayed production of ROS compared to other APEC lineages, but there was no significant (*p* ≤0.05) difference in ROS production between the isolates overall. Together, this is indicative that APEC clonal lineages have differing capacity to survive within avian macrophages, despite comparable immunological responses following infection.

### Virulence in Galleria mellonella is varied between APEC clonal groups

**Figure 6.**
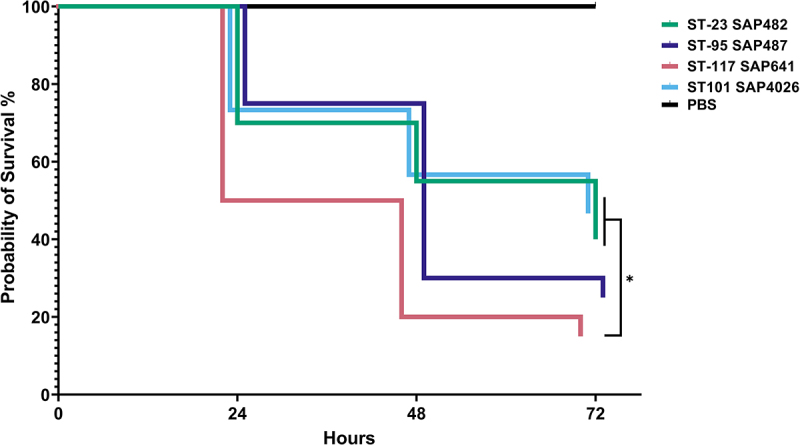
Survival of *Galleria mellonella* challenged with APEC clonal groups ST-23, ST-95, ST-117, and ST-101 24 hours, 48 hours, and 72 hours following challenge, expressed by Kaplan-Meier plots. Larvae challenged with 1x10^3^ CFU APEC or PBS mock and survival determined every 24 hours by confirming response to stimuli. Experiment performed with 10 larvae per group. Significance determined log-rank (Mantel-Cox) test *= *p ≥* 0.05.

*Galleria* larvae were used to compare the virulence of APEC clonal groups and establish whether enhanced survival observed in ST-101 and ST-95 correlated with a greater virulence potential. Larvae were challenged with selected isolates; ST-23 SAP482, ST-95 SAP487, ST-117 SAP641, and ST-101 SAP4026, with survival assessed as an indicator of virulence. Comparison of survival curves showed a trend (*p* = 0.0537) of differing larval mortality between all APEC isolates (Figure 6). Despite demonstrating comparatively reduced invasion and intramacrophage survival, the ST-117 SAP487 isolate induced significantly increased mortality with *Galleria* larvae at each timepoint examined compared to ST-23 SAP482 (*p* = 0.0487) and ST-101 SAP4026 (*p* = 0.011). The ST-95 SAP487 isolate also displayed a unique virulence trend, with increased mortality at 48 hours relative to SAP482 and SAP503. Collectively, this suggests that the virulence of APEC in a *Galleria* infection model differs between APEC clonal groups and may not be dependent on intracellular survival.

## Discussion

The APEC pathotype is highly diverse, with several genotypically distinct high-risk clonal groups identified [9,30]. Although these groups have been extensively characterized [9,13,40,41,57–60], how these genotypes may differ from one another phenotypically and manifest disease remains poorly understood. Here, ST-101 isolates identified were observed to encode a high density of virulence genes on a ColV plasmid which shares large regions of homology with previously reported APEC plasmids. Investigation of adhesion and invasion capacity of ST-101 APEC and major APEC lineages in the 8E11 chicken gut epithelial cell line revealed isolates belonging to ST-95, ST-140, and ST-101 possess higher invasive ability compared to ST-23 and ST-117. A similar relationship was also observed in the capacity of APEC isolates to survive intracellularly in the HD11 chicken macrophage cell line, with ST-23 and ST-117 demonstrating reduced survival compared to other sequence types examined. Infection of *Galleria mellonella* suggested that APEC lineages have varied virulence capability, and that ST-117 was the most virulent of all lineages tested despite reduced epithelial invasion and intramacrophage survival relative to other STs.

Genomic characterization of APEC from a colibacillosis outbreak in turkey poults identified multiple genotypes associated with disease (Figure 1). The majority (59.2%) of isolates identified with this study were found to belong to ST-101, ST-7804, ST-1011, and ST-3580, however with ST-101 was observed to be the dominant outbreak lineage, consistent with previous reports in chickens [52]. However, previous reports within turkeys have observed ST-101 to account for a minority of clinical isolates (0.3%) [29], or only within chicken isolates [61], and was not detected in other studies [30,62,63]. This is therefore the first study to report an outbreak in turkeys wherein ST-101 is the dominant lineage.

In addition to being the most frequently identified, the ST-101 lineage also demonstrated a strong association with disease state and lethality compared to other APEC genotypes detected. Asymptomatic carriage of APEC within the reservoir of the avian gut is well documented [15,17,19] and is typically considered to precede systemic opportunistic infections [17]. However, previous studies have demonstrated that there are significant differences in the genomic backgrounds and virulence of turkey cecal and clinical isolates [39]. This suggests that carriage of differing lineages is associated with differing disease risk, like what has been observed within this study between ST-101, and ST-7804/ST-1011.

The ST-101 isolates identified were observed to encode a high density of virulence genes on a ColV plasmid which shares large regions of homology with previously reported APEC plasmids. Investigation of adhesion and invasion capacity of ST-101 APEC and major APEC lineages in the 8E11 chicken gut epithelial cell line revealed isolates belonging to ST-95, ST-140, and ST-101 possess higher invasive ability compared to ST-23 and ST-117. A similar relationship was also observed in the capacity of APEC isolates to survive intracellularly in the HD11 chicken macrophage cell line, with ST-23 and ST-117 demonstrating reduced survival compared to other sequence types examined. Infection of *Galleria mellonella* suggested that APEC lineages have varied virulence capability, and that ST-117 was the most virulent of all lineages tested despite reduced epithelial invasion and intramacrophage survival relative to other STs.

Previous studies have aimed to characterize APEC outbreaks within poultry and documented the incidence of specific AMR and virulence genes [39,64–66], as well as the contribution to virulence by APEC associated plasmids [23,26]. Here, we identified ST-101 as an outbreak strain; this lineage has been detected globally and heavily associated with multidrug resistance (MDR) and pan-drug resistance phenotypes [67,68], human extraintestinal infection [69–74], as well as the environment [75]. Within birds, ST-101 was first identified in a colibacillosis outbreak in Spain [39], but has since been detected in Germany, Australia, Sweden, Brazil, and the UK [13,32,50,51]. It is therefore imperative the characteristics of this lineage are understood so that it can be managed effectively.

We have previously described the predominant APEC high-risk clonal groups ST-23 (phylogroup C), and ST-117 (phylogroup G) APEC are commonly reported in colibacillosis outbreaks [37–39], and comprise the most reported serotype in APEC infection, O78 [9]. Additionally, ST-95 and ST-140 APEC comprise two of the three distinct subpopulations within APEC-associated phylogroup B2 [9,40]. Within turkeys, the majority of clinical isolates belong to phylogroup B2, while cecal isolates are predominantly phylogroup B1 [30]. With ST-101 identified as belonging to phylogroup B1, this may suggest that this lineage may have a distinct evolutionary trajectory leading to a unique mechanism of action and eventual capacity to cause disease within birds. Therefore, cell culture models were utilized to investigate the mechanism of action of the ST-101 lineages, in comparison to high-risk clonal APEC clonal groups. Here, APEC sequence types were observed to differ in their invasive capacity. Invasion of host cells can provide several advantages to bacterial pathogens, providing access to nutrients and providing protection from immunological responses [53]. All lineages investigated demonstrated the ability to adhere to, and invade the gut epithelial cell line, a phenotype commonly associated with most *E. coli* pathotypes through the action of surface-binding proteins [76]. Isolates belonging to ST-95, ST-140, and ST-101 had comparatively higher levels of invasion within the 8E11 cell line relative to ST-23 and ST-117. The invasive ability of APEC has been previously investigated in 8E11 cells [77], as well as tracheal [78], lung [79,80], and liver epithelial cells [80], wherein invasion has been reported, mirroring what has been observed within this study. However, due to vast differences in cell type and the use of only a single APEC isolate, it is difficult to corroborate the observed differences between sequence type. However, comparison of an ST-101 O154 ExPEC isolated from a human patient to other ExPEC from phylogroup B2 highlighted an enhanced ability to invade epithelial in ST-101 ExPEC [68]. This contrasts with observations made in this study, where ST-101 APEC had similar invasive ability to isolates belonging to phylogroup B2 (ST-95 and ST-140). This discordance may be a result of differing virulence factors present between APEC clonal groups, with pronounced virulence gene profiles observed within previously characterized colibacillosis outbreaks [13,14], as well as APEC characterized within this study (Figure 1).

In addition to the invasion of gut epithelial cells, the ability of the representative APEC lineages to survive within the HD11 chicken macrophage cell line was examined. *E. coli* has been frequently demonstrated to resist macrophage killing and even proliferate following phagocytosis [55,81–86]. The ability of APEC to persist within HD11 cells has also been previously reported [87], similarly all APEC demonstrated the ability to survive at comparably low cell density beyond 18 hours post infection, but trends between intramacrophage survival and sequence type were observed. Alike the invasion of 8E11 cells, ST-95, ST101, and ST-140 demonstrated increased intracellular survival relative to ST-23 and ST-117. Enhanced intramacrophage survival of ST-140 and ST-95 isolates may be a result of the presence of the K1 capsule, typically associated with phylogroup B2 *E. coli* [88]. K1 capsules have been observed to confer resistance to phagocyte engulfment and killing in *E. coli* [89], Group B *Streptococcus* [90], and *Staphylococcus aureus* [91]. Previous studies have also investigated the interactions of APEC strains with phagocytes, with Mellata *et al*. observing reduced association with, and phagocytic bactericidal activity against, serotype O78 APEC (assumed to be ST-23 or ST-117) relative to O1 or O2 APEC in primary macrophages and heterophils [92]. In contrast, no reduction in association was observed within this study with O78 APEC, in addition to increased bacterial clearance. This may be a result of differing genomic background between isolates, opposed to O-antigen identity, with Mellata *et al*. also observing increased type 1 fimbriae expression enhancing resistance of phagocyte killing. Furthermore, additional studies have observed a continuous reduction in O78 APEC intramacrophage survival [87], while other work showed an O2 isolate had improved survival and demonstrated the ability to replicate within the same cell line [93].

Within macrophages, nitric oxide production reflects macrophage activation and ROS production is a valid measure of respiratory burst following activation. NO and ROS are involved in bacterial killing by the disruption of bacterial cellular processes through DNA damage and protein denaturation [56]. No significant difference in NO or ROS production following challenge with different APEC lineages was observed, suggesting that differences in intramacrophage survival are independent of these processes.

The *Galleria mellonella* infection model has been used frequently as an indicator of microbial virulence [46,47,94,95] and has recently been demonstrated to be a viable model for determining virulence in APEC, correlating with virulence assays performed in one-day old chicks [96]. The larval innate immune system also has functional similarities to that of vertebrates [97], allowing investigation of innate immune cell-pathogen interactions. Moreover, previous investigations have shown that virulence of ExPEC isolates within the *Galleria mellonella* infection model differs between sequence types [98,99]. Similarly, this study presents differences in virulence potential between APEC sequence types, with SAP641 ST-117 showing greatest virulence potential. ST-117 APEC belong to phylogroup G and have emerged as the most frequently implicated cause of colibacillosis associated with high pathogenicity [6,30,65,100]. Therefore, it is unsurprising that this lineage displays high levels of virulence with *Galleria* larvae. This high *in vivo* virulence potential may appear inconsistent with observed phenotypic interactions within 8E11 and HD11 cells, wherein ST-117 survival was low. However, use of a whole organism model with separate tissue compartments and a multi-layered immune response that encompasses coagulation, encapsulation, and melanization, are not directly comparable to cell that only model cell-autonomous innate immunity. Our use of a combination of models has built a broader picture of immunomodulation and made clear that the capacity to invade and survive intracellularly are not the only process governing virulence potential of APEC. Moreover, our results suggest distinct host-pathogen interactions between APEC clonal groups which require further investigation.

Further investigation characterizing the phenotypic nature of APEC genotypes is necessary to elucidate the determinants of these distinct behaviors outside of epithelial and macrophage interactions, as modeled with *Galleria mellonella*. However, as the *Galleria* model is limited by the lack of an adaptive immune system, future studies may provide greater insight into the comparison of *in vivo* interactions of APEC clonal groups using alternative models such as day-old chick models or embryo lethality assays [101,102]. The use of one-day old chick models would also facilitate the comparison of APEC clonal groups via different infection routes such as [103]. This may reveal novel interactions within APEC genotypes, which cannot be modeled with the 8E11 and HD11 cell lines alone.

In summary, we present evidence to suggest that pronounced differences in phenotypic interactions with host cells and virulence are present between high-risk APEC clonal groups. This provides further evidence that the genotypes most commonly implicated in colibacillosis exhibit considerable heterogeneity with regards to their pathogenic potential. Future investigations into the genetic determinants that govern phenotypic variation between these genotypes are essential to inform potential future disease management strategies.

## Supplementary Material

Figure_S1_virulence_.jpg

Figure S2- QVIR-2025-0124.R1.jpg

Clean copy of Supplementary figures - QVIR-2025-0124.R1.docx

Table_S1.docx

## Data Availability

The authors confirm all raw data and supplementary files have been provided within the article or are available in the public repository Zenodo DOI: 10.5281/zenodo.14965605, https://zenodo.org/records/14965605. Genome sequence data are available within the Genomes repository at NCBI, in Bioproject Accession PRJNA1222627. The information for the APEC genomes has been included in Supplementary File 1.
