# Supplementary figures and images for: High-risk clonal groups of avian pathogenic Escherichia coli (APEC) demonstrate heterogeneous phenotypic characteristics in vitro and in vivo
Source: Virulence. 2025 Aug 13;16(1):2546682. doi: 10.1080/21505594.2025.2546682 (PMC12351747; doi:10.1080/21505594.2025.2546682)

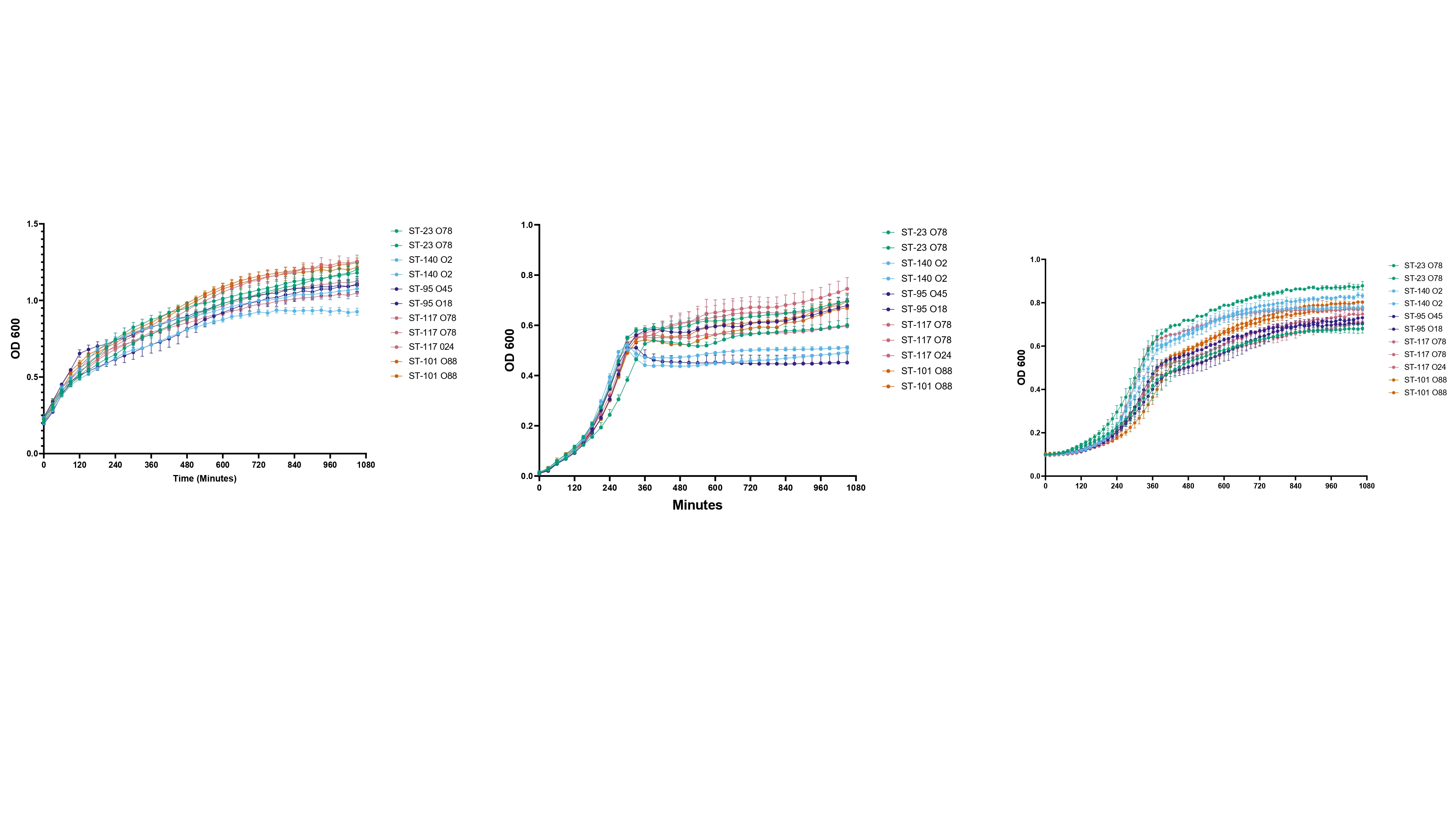

Supplement: Figure_S1_virulence_.jpg [file KVIR_A_2546682_SM9303.jpg]

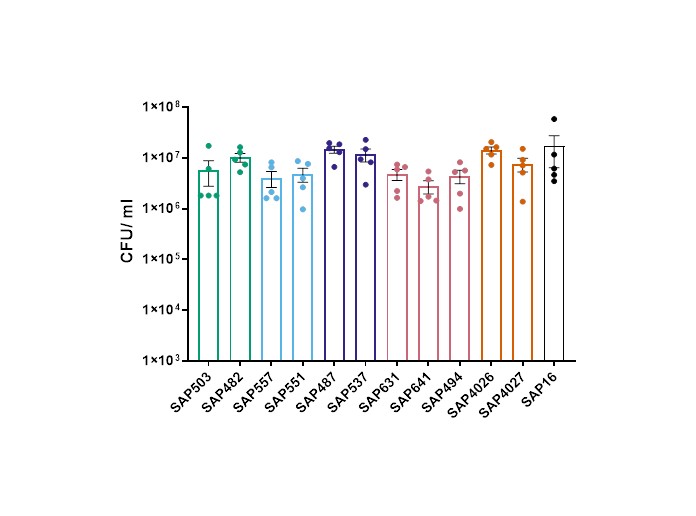

Supplement: Figure S2- QVIR-2025-0124.R1.jpg [file KVIR_A_2546682_SM9302.jpg]
