# Supplementary material for: High-risk clonal groups of avian pathogenic Escherichia coli (APEC) demonstrate heterogeneous phenotypic characteristics in vitro and in vivo
Source: Virulence. 2025 Aug 13;16(1):2546682. doi: 10.1080/21505594.2025.2546682 (PMC12351747; doi:10.1080/21505594.2025.2546682)
Supplement: Table_S1.docx [file KVIR_A_2546682_SM9300.docx]

**Table S1**: List of APEC isolates and a single plasmid that were sequenced as part of this study. All isolates were derived from heart, liver or caecal contents of sick or healthy turkey poults and confirmed as E. coli. The Biosample accession, date of isolation, anatomical site of isolation, bird status, and sequence type are displayed.

| **APEC isolate name** | **BioSample Accession** | **Date of isolation** | **Host** | **Anatomical site** | **Diseased/Healthy** | **Sequence Type** |
| --- | --- | --- | --- | --- | --- | --- |
| pSAP4027 | SAMN46824902 | N/A | N/A | N/A | N/A | N/A |
| SAP4026 | SAMN46824900 | 14/06/2019 | Turkey | Heart | S | 101 |
| SAP4027 | SAMN46824901 | 14/06/2019 | Turkey | Caeca | S | 101 |
| SAP4442 | SAMN46824810 | 08/05/2019 | Turkey | Caeca | H | 429 |
| SAP4443 | SAMN46824811 | 20/06/2019 | Turkey | Caeca | S | 58 |
| SAP4444 | SAMN46824812 | 08/05/2019 | Turkey | Caeca | S | 429 |
| SAP4445 | SAMN46824813 | 08/05/2019 | Turkey | Caeca | H | 429 |
| SAP4446 | SAMN46824814 | 09/09/2019 | Turkey | Caeca | H | None identified |
| SAP4447 | SAMN46824815 | 18/09/2019 | Turkey | Heart | S | 429 |
| SAP4448 | SAMN46824816 | 08/05/2019 | Turkey | Heart | S | 429 |
| SAP4449 | SAMN46824817 | 08/05/2019 | Turkey | Heart | S | 429 |
| SAP4450 | SAMN46824818 | 08/05/2019 | Turkey | Heart | S | 429 |
| SAP4451 | SAMN46824819 | 08/05/2019 | Turkey | Caeca | H | 1161 |
| SAP4452 | SAMN46824820 | 08/05/2019 | Turkey | Caeca | H | 1161 |
| SAP4453 | SAMN46824821 | 08/05/2019 | Turkey | Caeca | H | 1161 |
| SAP4454 | SAMN46824822 | 14/06/2019 | Turkey | Caeca | S | 101 |
| SAP4455 | SAMN46824823 | 14/06/2019 | Turkey | Caeca | H | None identified |
| SAP4456 | SAMN46824824 | 16/09/2019 | Turkey | Caeca | H | 7804 |
| SAP4457 | SAMN46824825 | 20/06/2019 | Turkey | Liver | S | 101 |
| SAP4458 | SAMN46824826 | 20/06/2019 | Turkey | Caeca | S | 101 |
| SAP4459 | SAMN46824827 | 20/06/2019 | Turkey | Liver | S | 101 |
| SAP4460 | SAMN46824828 | 14/06/2019 | Turkey | Heart | S | 6949 |
| SAP4461 | SAMN46824829 | 14/06/2019 | Turkey | Heart | S | 6949 |
| SAP4462 | SAMN46824830 | 14/06/2019 | Turkey | Heart | S | 6949 |
| SAP4463 | SAMN46824831 | 20/06/2019 | Turkey | Heart | S | 101 |
| SAP4465 | SAMN46824833 | 16/09/2019 | Turkey | Liver | S | 7804 |
| SAP4466 | SAMN46824834 | 20/06/2019 | Turkey | Heart | S | 101 |
| SAP4467 | SAMN46824835 | 20/06/2019 | Turkey | Heart | S | 101 |
| SAP4468 | SAMN46824836 | 20/06/2019 | Turkey | Caeca | S | 373 |
| SAP4469 | SAMN46824837 | 20/06/2019 | Turkey | Caeca | S | 5926 |
| SAP4470 | SAMN46824838 | 20/06/2019 | Turkey | Caeca | S | 101 |
| SAP4471 | SAMN46824839 | 20/06/2019 | Turkey | Caeca | S | 101 |
| SAP4472 | SAMN46824840 | 18/06/2019 | Turkey | Caeca | S | 12 |
| SAP4473 | SAMN46824841 | 20/06/2019 | Turkey | Heart | S | 101 |
| SAP4474 | SAMN46824842 | 20/06/2019 | Turkey | Liver | S | 101 |
| SAP4475 | SAMN46824843 | 18/06/2019 | Turkey | Caeca | S | 3580 |
| SAP4476 | SAMN46824844 | 20/06/2019 | Turkey | Caeca | S | 101 |
| SAP4477 | SAMN46824845 | 14/06/2019 | Turkey | Caeca | H | 101 |
| SAP4478 | SAMN46824846 | 20/06/2019 | Turkey | Heart | S | 101 |
| SAP4479 | SAMN46824847 | 20/06/2019 | Turkey | Liver | S | 101 |
| SAP4480 | SAMN46824848 | 14/06/2019 | Turkey | Heart | S | 101 |
| SAP4481 | SAMN46824849 | 20/06/2019 | Turkey | Caeca | S | 101 |
| SAP4482 | SAMN46824850 | 08/05/2019 | Turkey | Caeca | S | 58 |
| SAP4483 | SAMN46824851 | 20/06/2019 | Turkey | Liver | S | 101 |
| SAP4484 | SAMN46824852 | 20/06/2019 | Turkey | Caeca | S | 101 |
| SAP4485 | SAMN46824853 | 14/06/2019 | Turkey | Caeca | S | 101 |
| SAP4486 | SAMN46824854 | 18/09/2019 | Turkey | Heart | S | 101 |
| SAP4487 | SAMN46824855 | 14/06/2019 | Turkey | Caeca | S | 7804 |
| SAP4488 | SAMN46824856 | 20/06/2019 | Turkey | Caeca | S | 101 |
| SAP4489 | SAMN46824857 | 09/09/2019 | Turkey | Liver | S | 1011 |
| SAP4490 | SAMN46824858 | 09/09/2019 | Turkey | Caeca | H | 7804 |
| SAP4491 | SAMN46824859 | 09/09/2019 | Turkey | Caeca | H | 1011 |
| SAP4492 | SAMN46824860 | 09/09/2019 | Turkey | Heart | S | 1011 |
| SAP4493 | SAMN46824861 | 09/09/2019 | Turkey | Liver | S | 1011 |
| SAP4494 | SAMN46824862 | 16/09/2019 | Turkey | Caeca | H | 7804 |
| SAP4495 | SAMN46824863 | 09/09/2019 | Turkey | Caeca | H | 1011 |
| SAP4496 | SAMN46824864 | 09/09/2019 | Turkey | Caeca | H | 1011 |
| SAP4497 | SAMN46824865 | 09/09/2019 | Turkey | Heart | S | 1011 |
| SAP4498 | SAMN46824866 | 09/09/2019 | Turkey | Caeca | H | 1011 |
| SAP4499 | SAMN46824867 | 09/09/2019 | Turkey | Caeca | H | 942 |
| SAP4500 | SAMN46824868 | 16/09/2019 | Turkey | Liver | S | 7804 |
| SAP4501 | SAMN46824869 | 20/06/2019 | Turkey | Liver | S | 7804 |
| SAP4502 | SAMN46824870 | 16/09/2019 | Turkey | Caeca | H | 373 |
| SAP4503 | SAMN46824871 | 09/09/2019 | Turkey | Caeca | H | 7804 |
| SAP4504 | SAMN46824872 | 16/09/2019 | Turkey | Caeca | H | 7804 |
| SAP4505 | SAMN46824873 | 09/09/2019 | Turkey | Caeca | H | 7804 |
| SAP4506 | SAMN46824874 | 08/05/2019 | Turkey | Caeca | S | 373 |
| SAP4507 | SAMN46824875 | 16/09/2019 | Turkey | Caeca | H | 7804 |
| SAP4508 | SAMN46824876 | 16/09/2019 | Turkey | Caeca | H | 7804 |
| SAP4509 | SAMN46824877 | 16/09/2019 | Turkey | Caeca | S | 685 |
| SAP4510 | SAMN46824878 | 18/09/2019 | Turkey | Caeca | H | 349 |
| SAP4511 | SAMN46824879 | 18/09/2019 | Turkey | Caeca | S | 3580 |
| SAP4512 | SAMN46824880 | 18/09/2019 | Turkey | Caeca | H | 602 |
| SAP4513 | SAMN46824881 | 18/09/2019 | Turkey | Caeca | S | 3580 |
| SAP4514 | SAMN46824882 | 18/09/2019 | Turkey | Caeca | S | 3580 |
| SAP4515 | SAMN46824883 | 18/09/2019 | Turkey | Heart | S | 12 |
| SAP4516 | SAMN46824884 | 18/09/2019 | Turkey | Caeca | H | 3580 |
| SAP4517 | SAMN46824885 | 18/09/2019 | Turkey | Heart | S | 10 |
| SAP4518 | SAMN46824886 | 18/09/2019 | Turkey | Heart | S | 10 |
| SAP4519 | SAMN46824887 | 20/06/2019 | Turkey | Caeca | H | 12 |
| SAP4520 | SAMN46824888 | 18/09/2019 | Turkey | Heart | S | 3580 |
| SAP4521 | SAMN46824889 | 18/09/2019 | Turkey | Caeca | H | 602 |
| SAP4522 | SAMN46824890 | 16/09/2019 | Turkey | Heart | S | 602 |
| SAP4523 | SAMN46824891 | 16/09/2019 | Turkey | Liver | S | 355 |
| SAP4524 | SAMN46824892 | 18/09/2019 | Turkey | Heart | S | 3580 |
| SAP4525 | SAMN46824893 | 18/09/2019 | Turkey | Caeca | S | 3258 |
| SAP4526 | SAMN46824894 | 18/09/2019 | Turkey | Heart | S | 12 |
| SAP4527 | SAMN46824895 | 18/09/2019 | Turkey | Caeca | S | 3258 |
| SAP4528 | SAMN46824896 | 18/09/2019 | Turkey | Caeca | S | 12 |
| SAP4529 | SAMN46824897 | 18/09/2019 | Turkey | Caeca | S | 12 |
| SAP4530 | SAMN46824898 | 18/09/2019 | Turkey | Caeca | H | 101 |
| SAP4531 | SAMN46824899 | 18/09/2019 | Turkey | Caeca | H | 429 |
